# Pneumonitis Induced by Immune Checkpoint Inhibitors: From Clinical Data to Translational Investigation

**DOI:** 10.3389/fonc.2020.01785

**Published:** 2020-09-11

**Authors:** Shicong Zhu, Yang Fu, Bo Zhu, Bicheng Zhang, Jun Wang

**Affiliations:** ^1^Cancer Center, Renmin Hospital of Wuhan University, Wuhan, China; ^2^Department of Oncology, Xiangyang Hospital, Hubei University of Chinese Medicine, Xiangyang, China; ^3^Department of Oncology, Xinqiao Hospital, Army Medical University, Chongqing, China; ^4^Department of Oncology, The First Affiliated Hospital of Shandong First Medical University, Jinan, China

**Keywords:** immune-related adverse events, programmed cell death 1, programmed cell death ligand 1, immune checkpoint inhibitor, pneumonitis

## Abstract

Immune checkpoint inhibitors (ICIs) have been applied to clinical practice and achieved significant therapeutic benefit in a variety of human malignancies. These drugs not only enhance the body’s antitumor immune response but also produce side effects called immune-related adverse events (irAEs). Although checkpoint inhibitor pneumonitis (CIP) has a low clinical incidence, it is likely to cause the delay or termination of immunotherapy and treatment-related death in some severe cases. An increasing number of CIP cases have been reported since 2015, which are attributed to the augmentation of approvals and uses of ICIs, but a comprehensive understanding of CIP is still lacking. This review focuses on the epidemiology, clinical characteristics, treatment strategies, and underlying mechanisms of CIP to strengthen the recognition of pulmonary toxicity among clinicians and researchers.

## Introduction

Immune checkpoint inhibitors (ICIs) can restore the body’s antitumor immune response and promote T cell-mediated clearance of tumor cells by blocking the inhibitory signaling pathways of T cells (1). As immune checkpoints work, the inhibitory signals are mediated by programmed cell death 1 (PD-1) binding to its two specific ligands, programmed cell death ligand 1 (PD-L1), and programmed cell death ligand 2 (PD-L2) expressed on tumor cells, as well as cytotoxic T lymphocyte-associated protein 4 (CTLA-4), binding to B7-1 (CD80), and B7-2 (CD86) molecules on antigen-presenting cells (APCs).

In recent years, immunotherapy with ICIs, consisting of PD-1 inhibitors, PD-L1 inhibitors and CTLA-4 inhibitors, has become an important therapeutic strategy for advanced malignant tumors. Two PD-1 inhibitors (nivolumab and pembrolizumab), three PD-L1 inhibitors (atezolizumab, durvalumab, and avelumab), and one CTLA-4 inhibitor (ipilimumab) have been approved by the US Food and Drug Administration (FDA) for multiple types of malignancies, mainly containing advanced melanoma, non-small cell lung cancer (NSCLC), and renal cell carcinoma (RCC). Over 50 immunotherapy agents are under drug research and development in the United States, and more than 800 clinical studies for tumor immunotherapy are ongoing (2).

With the wide application of these drugs, immune-related adverse events (irAEs) have also increased, mainly including fatigue, skin toxicity, colitis, hepatitis, thyroiditis, and pneumonitis (3). The degrees of irAEs are mostly from mild to moderate, but there are also serious adverse reactions that endanger patients’ lives, such as immune-related pneumonitis, nephritis, and myocarditis. Pneumonitis induced by ICIs is now referred to as checkpoint inhibitor pneumonitis (CIP) (4). Although CIP is rare, it has a poor prognosis, accounting for 28% of fatal events (5). More and more cases of CIP have been reported in recent years, but knowledge about it remains limited. In this review, the clinical features of CIP and related translational investigations will be discussed.

## Incidence and Risk Factors

A meta-analysis containing 26 studies showed that the overall incidence of CIP was 2.7% for all grades and 0.8% for grade 3 or above (6). About 0.2% of patients died from pneumonitis, and 0.2% to 4.0% of patients discontinued the PD-1 inhibitors due to pneumonitis (6). It is worth noting that a recent research in patients with NSCLC suggested that the incidence of CIP seemed higher in the real world, with an all-grade incidence rate of 19% and a high-grade (grade 3 or 4) incidence rate of 11% (7). The data in meta-analysis and multicenter clinical trials are shown in Table 1 (6, 8–12).

**TABLE 1:** Incidence of CIP.

Study author	Numbers of		Tumor type	ICIs	Incidence of		
	Trials	Patients			All grade	Grade 3/4	Pneumonitis-related death
Nishino et al. (6)	20	4,496	Melanoma, NSCLC, RCC, etc.	Nivolumab, pembrolizumab, and ipilimumab	2.7%	0.8%	0.2–2.3%
Abdel-Rahmen et al. (8)	11	6,671	Melanoma, NSCLC, RCC, prostate cancer	Nivolumab, pembrolizumab, and ipilimumab	1.3–11%	0.3–2.0%	–
Costa et al. (9)	9	5,353	Melanoma, NSCLC, RCC, etc.	Nivolumab, pembrolizumab, and ipilimumab	2.65%	–	–
Nishijima et al. (10)	7	3,450	Melanoma, NSCLC	Nivolumab, pembrolizumab, and atezolizumab	3.4%	1.3%	–
Delaunay et al. (11)	–	1,826	Melanoma, NSCLC	CTLA-4, PD-1, and PD-L1 inhibitors	3.5%	1.26%	0.33%
Naidoo et al. (12)	–	915	Melanoma, NSCLC, RCC, etc.	CTLA-4, PD-1, and PD-L1 inhibitors	4.7%	1.2%	0.1%

*CIP, checkpoint inhibitor pneumonitis; ICIs, immune checkpoint inhibitors; NSCLC, non-small-cell lung cancer; RCC, renal cell carcinoma; CTLA-4, cytotoxic T lymphocyte-associated protein 4; PD-1, programmed cell death protein 1; and PD-L1, programmed cell death-ligand 1.*

However, it is different in terms of the incidence within different drugs. Due to the different toxicity profiles of ICIs, PD-1 inhibitors were more likely to induce the CIP than CTLA-4 inhibitors (OR 6.4, 95% CI 3.2–12.7) (13). In addition, the toxicity profiles of the PD-1 and PD-L1 inhibitors may also be different. A meta-analysis showed that the incidence of all-grade pneumonitis relative to PD-1 inhibitors was higher than PD-L1 inhibitors (3.6% vs. 1.3%), also for grades 3 and 4 (1.1% vs. 0.4%) (14). Likewise, the incidence of CIP caused by different PD-1 inhibitors did not seem to be precisely the same. The patients treated with pembrolizumab were more likely to experience pneumonitis for all grades than the patients treated with nivolumab (OR 2.08, 95% CI 1.52–2.85), but there was no significant difference for high grades between these two drugs. In addition, the study showed that atezolizumab and nivolumab or atezolizumab and pembrolizumab had no significant difference regarding the incidence of pneumonitis at all grades and higher (15).

Moreover, the incidence of CIP varied in different tumor types. According to a systematic review, pneumonitis appeared more likely to occur in NSCLC or RCC patients (6). Studies showed that the incidence of pneumonitis in NSCLC patients was significantly higher than that in melanoma patients for both all grades (4.1% vs. 1.6%) and higher grades (1.8% vs. 0.2%). However, the odds of all-grade pneumonitis were higher in RCC than melanoma (4.1% vs. 1.6%) but have no difference in grade 3 or higher (0.8% vs. 0.2%) (6, 15), although in the same type of tumor, different pathological types seemed to have an impact on the incidence of CIP. Data from a retrospective study indicated that adenocarcinoma tumor histology was associated with a lower risk of CIP compared with non-adenocarcinoma histology (including squamous NSCLC; OR 0.38, 95% CI 0.17–0.82) (5).

Furthermore, the incidence of pneumonitis in combination therapy and monotherapy was also different. In the checkmate227 study, the incidence of all-grade (3.8% vs. 2.3%), or grade 3–4 pneumonitis (2.3% vs. 1.5%) in nivolumab plus ipilimumab group was higher than that in the nivolumab monotherapy group (16). A meta-analysis compared the incidence of CIP among different therapeutic regimens in melanoma, and the result showed that combination therapy had a higher incidence of CIP than PD-1 inhibitor monotherapy for all grades (6.6% vs. 1.6%) and grade 3 or higher (1.5% vs. 0.2%) (6). The combination described above included a combination of dual ICIs and ICIs plus peptide vaccines. Subsequently, another meta-analysis including melanoma, NSCLC, small cell lung cancer, and other tumor types indicated that the risk of all-grade CIP (3.47 times) and severe CIP (3.48 times) was higher in combination therapy (ipilimumab plus nivolumab) than nivolumab or ipilimumab alone (17). However, there are no data on the incidence of CIP in ICIs plus chemotherapy vs. ICI monotherapy.

Besides, researchers are also concerned about many other related risk factors for CIP. One study showed that patients with a history of asthma/chronic obstructive pulmonary disease (COPD; 5.4% vs. 3.1%) or who had previously received chest radiotherapy (6.0% vs. 2.6%) were more susceptible to CIP than those without COPD or chest radiotherapy, respectively (18). Some studies also manifested that high-risk populations for CIP included those with NSCLC possessing sensitizing epidermal growth factor receptor (EGFR) mutation when treated with EGFR–tyrosine kinase inhibitor (TKI) in combination with ICIs, and those with an active lung infection (7, 19, 20). Kato et al. pointed out that male gender, smoking history, and early multiline treatment were the potential risk factors for pneumonitis caused by nivolumab (21), although some studies did not consider gender as a risk factor (6). Naidoo et al. found that smoking and baseline lung disease were not only the potential risk factors of CIP but also related to poor response to steroid therapy for CIP (12). Interestingly, research indicated that extrathoracic metastasis was associated with a significantly lower incidence of CIP (22). However, the occurrence of CIP caused by PD-1 inhibitors seemed to have no significant relationship with the dose of ICIs and the age of the patients (23).

The relationship between the occurrence of CIP and immunotherapy efficacy is also one of the concerns of researchers. Several studies reported that the occurrence of irAEs was related to a better efficacy or even survival outcome in patients treated with ICIs (24, 25). However, as one of many irAEs, whether CIP can also be taken for an excellent prognostic indicator remains a question. A multi-institutional analysis suggested that the development of pneumonitis was significantly associated with increased progression-free survival (PFS) and overall survival (OS) in patients with advanced NSCLC treated with nivolumab (26). Nevertheless, some other studies found that treatment efficacy and survival were significantly decreased in patients with CIP compared with those without ICI therapy in NSCLC (5, 27, 28), while another retrospective study showed that no significant survival differences were seen with the occurrence of pneumonitis in metastatic melanoma treated with nivolumab (29).

## Clinical Presentation

The time from the administration of ICIs to the occurrence of CIP varied from 2 to 24 months, with a median time of 2.8 months (12). Moreover, studies reported that high-grade CIP occurred earlier than low-grade CIP (7).

There was no difference in onset time between different ICIs, but patients with combination therapy seemed to have an earlier onset of CIP (median, 2.7 vs. 4.6 months). A retrospective study indicated that patients with NSCLC developed pneumonitis earlier than patients with malignant melanoma (median, 2.1 vs. 5.2 months) (11).

The main clinical symptoms of CIP include dyspnea (53%), cough (35%), fever (12%), and chest pain (7%) (30). Most patients with CIP had mild symptoms, with grade 1–2 CIP accounting for about 73% (30). It is worth noting that recurrent pneumonitis was usually more severe than the first event (31). However, there was no difference in the distribution of severity between monotherapy and combination therapy (12). In addition, approximately 25% of patients have other immune-related symptoms at the same time or have no symptoms.

The radiographic features of CIP are diverse and non-specific. Most can be shown as traction bronchiectasis, consolidation, reticular opacities, ground-glass opacity (GGO), centrilobular nodularity, and honeycombing (32). Naidoo et al. summarized radiologic features as five subtypes: cryptogenic organizing pneumonia (COP) like (19%), mainly manifested as discrete patchy or confluent shadows with or without air bronchography; GGO (37%), mainly manifested as frosted glass-like nodules in the periphery or under the pleura; non-specific interstitial pneumonia (NSIP; 7%), chest CT showed thickened lobular septa, infiltrated around the bronchial blood vessels, and severe cases showed a subpleural mesh or honeycomb structure; hypersensitivity pneumonitis (HP; 22%), mainly manifested as nodules in the center of the leaflets or bronchiole-like appearance of tree-like micro-nodules; and others (15%) (12). Moreover, acute interstitial pneumonia (AIP), and acute respiratory distress syndrome (ARDS) have also been reported (33). In addition to the typical manifestations of pneumonia, some case reports suggested the presence of small subpleural nodules, hilar lymphadenopathy, and granulomatous changes (12). According to previous research reports, the radiologic subtypes are consistent throughout the patients’ clinical course, with a few exceptions, including the evolution from COP-like subtype to severe GGO type and the additional interstitial appearance of GGO type (12).

In clinical practice, the differential diagnosis of CIP is of considerable significance, but it often cannot be definitively diagnosed by imaging alone. Firstly, CIP often needs to be distinguished from infectious pneumonia, including bacteria, viruses, tuberculosis, and fungi. Infected patients usually have symptoms of fever, sputum, and elevated white blood cells. Compared with infectious pneumonia, CIP is less prone to fever and more prone to respiratory failure (34). The imaging manifestation of infectious pneumonia is ground-glass shadow in the early stage, bacterial pneumonia lesions are limited to lung lobes or lung segments, and viral pneumonia can be multiple ground-glass shadows. Lung consolidation may occur after the disease progresses. A combination of bronchoscopy and various etiological examinations (such as nasal swab, blood culture, sputum culture, and urine culture) may help exclude infection (35). Furthermore, tumor progression that leads to new lesions also needs to be identified with CIP. The clinical manifestations of tumor progression are cough, hemoptysis, chest pain, weight loss, dyspnea, and cough. Besides, serum tumor markers are often higher than before. Imaging manifestations of the progression of the primary lesion of lung cancer are often an increase in the primary lesion of lung cancer and new nodular shadows, patchy shadows, ground-glass shadows. Imaging of lung cancer lymphangitis due to progress is characterized by the thickening of multiple leaflet septa and multiple small nodules. Radiation pneumonia most often occurs between 2 and 6 months after lung radiotherapy. Most of the lesions are confined to the radiation field, with or without respiratory symptoms, and symptoms may include cough, dyspnea, and low fever. Bronchoalveolar lavage (BAL) can be used in the differential diagnosis, often manifested by an increase in the proportion of lymphocytes. In addition, a prospective observational study suggested that lung function tests during treatment might be helpful for risk stratification to screen for CIP (36). Usually, a bronchoscopic biopsy is not considered, but it can be used when it is difficult to make a differential diagnosis (37).

## Clinical Management

Clinically, the treatment of CIP is carried out according to the principle of classification (38). Clinical classification of CIP refers to the National Cancer Institute (NCI) Common Terminology Criteria for Adverse Events (CTCAE) Version 4.03 and European Society for Medical Oncology (ESMO) Guidelines for Immunotherapeutic Toxicity Management (37, 39). However, the use of CTCAE still has some limitations on toxicity grading, sometimes underestimating or overestimating the probability and severity of toxicities (40).

According to the range of clinical symptoms and lesions involved, the guidelines classify toxicity into five grades: G1, mild toxicity; G2, moderate toxicity; G3, severe toxicity; G4, life-threatening toxicity; and G5, death-related toxicity (41). The classification description of CIP is shown in Table 2. For the management of G1 toxicities, closely observe the patient’s condition, repeat CT, and monitor the lung capacity in 3 to 4 weeks. Baseline examinations for CIP patients include chest CT, blood oxygen saturation, blood routine, liver and kidney function, electrolytes, erythrocyte sedimentation rate (ESR), C-reactive protein (CRP), and lung function. If improvement is observed, continue to follow up; if no improvement is observed, stop using ICIs and treat as G2. For the management of G2 toxicities, continue to stop using ICIs until there is improvement to G1 or less. Administer prednisone 1 to 2 mg/kg/day by intravenous drip. If improvement is observed, taper by 5 to 10 mg/week over 4 to 6 weeks; if no improvement is observed, treat as G3∼G4. For the management of G3∼G4 toxicities, permanently stop using ICIs. Administer methylprednisolone 2 mg/kg/day by intravenous injection. After steroid treatment for 48 h, if improvement is observed, the treatment continues until there is improvement to G1 or less, and taper corticosteroids over 4 to 6 weeks; if no improvement is observed, consider administering infliximab 5 mg/kg by intravenous drip, or mycophenolate mofetil (MMF) 1 *g* twice a day or immunoglobulin by intravenous injection. The management of CIP is described in Table 3.

**TABLE 2:** Gradation of CIP.

Grades	Description
G1	No symptom
	Limited to a single lobe or <25% lung parenchyma
G2	New symptoms or worsening symptoms, including shortness of breath, cough, chest pain, fever, and anoxia
	Involves multiple lung lobes and reaches 25–50% of lung parenchyma, affecting daily life, requiring drug intervention
G3	Serious new complications
	Involves all lung lobes or >50% of lung parenchyma, limited personal self-care ability, requiring oxygen inhalation and hospitalization
G4	Life-threatening dyspnea, acute respiratory distress syndrome (ARDS) requiring urgent intervention such as intubation

*CIP, checkpoint inhibitor pneumonitis.*

**TABLE 3:** Management of CIP.

Grades	Guideline for the management
G1	• Consider holding ICIs Monitor symptoms every 2–3 days
	• May offer one repeat CT in 3–4 weeks
	• In patients who have had baseline testing, may offer a repeat spirometry/DLCO in 3–4 weeks
	○ If improvement is observed, continue to follow up
	○ If condition worsens, treat as G2 or 3–4
G2	• Hold ICIs until resolution to G1 or less
	• Consider infectious workup: nasal swab for potential viral pathogens sputum culture, blood culture, and urine culture
	• Consider chest CT with contrast Repeat chest CT in 3–4 weeks
	• Consider empirical antibiotics if infection has not yet been fully excluded
	• Prednisone IV 1–2 mg/kg/day
	○ If improvement is observed, start slow steroid taper by 5 to 10 mg/week over 4 to 6 weeks
	○ If condition worsens, treat as G3–4
G3/G4	• Permanently discontinue ICIs
	• Pulmonary consultation for bronchoscopy with BAL
	Consider biopsies for atypical lesions Methylprednisolone IV 2–4 mg/kg/day
	○ If improvement is observed, taper corticosteroids over 4–6 weeks
	○ If not improving or worsening after 48 h: add infliximab IV 5 mg/kg
	or MMF IV 1 g BID
	or IVIG for 5 days
	or cyclophosphamide

*ICIs, immune checkpoint inhibitors; CT, computed tomography; DLCO, carbon monoxide diffusing capacity; IV, intravenous; BAL, bronchoalveolar lavage; MMF, mycophenolate mofetil; BID, two times daily; and IVIG, intravenous immunoglobulin.*

Steroid therapy is the most basic treatment for CIP. Regularly, adequate steroids can control 70–80% of CIP (35). Other treatments include infliximab, cyclophosphamide, MMF, tocilizumab, and immunoglobulin. The major guidelines are relatively uniform for the dosage of steroids in G2 (1–2 mg/kg/day), but when dealing with G3∼G4, the recommended dose in ESMO is higher than that of other guidelines (2–4 vs. 1–2 mg/kg/day). Regarding the overall course of steroid use, similarly, the opinions of the guidelines are relatively uniform in G2, and it is recommended that the overall course of treatment should be controlled within 4 weeks. However, as for G3∼G4, ESMO and Society for Immunotherapy of Cancer (SITC) emphasize that the process of steroid reduction should be slower. The recommended total course of treatment is 8 weeks in ESMO and SITC but 4–6 weeks in American Society of Clinical Oncology (ASCO) and National Comprehensive Cancer Network (NCCN).

It is worth noting that steroids and antibiotics are often used in CIP patients, but there seems to be a specific relationship between these two types of drugs and the efficacy of immunotherapy. The effect of using steroids on the survival of patients receiving ICI treatment is not entirely certain. A retrospective study showed that the patients who received prednisone >10 mg at the start of immunotherapy had a shorter median OS than those who received 0–10 mg of prednisone (4.9 vs. 11.2 months) (42). However, a recent meta-analysis pointed out that the use of steroids to mitigate adverse events did not negatively affect OS (43). Moreover, some studies showed that the use of antibiotics often leads to worse treatment response and OS in patients treated with ICIs (44, 45). Therefore, it is still necessary to be cautious when using steroids and antibiotics in CIP patients.

Patients with no clinical improvement after 48 to 72 h of corticosteroid therapy are considered to be steroid resistant. The evaluation of clinical signs and symptoms can include assessment of general condition, change in dyspnea or cough, and need for supplemental oxygen. Comprehensive judgment can be combined with objective indicators such as oxygen saturation and blood gas analysis. If necessary, review chest CT or chest radiograph to make a judgment. For these steroid-refractory CIP patients, it is recommended to consider administrating infliximab, MMF, or immunoglobulin as described above, but there is no consensus on the optimal choice and usage. Guacimara et al. reported that a case of mycophenolate-resistant CIP was successfully treated with infliximab, and they thought that infliximab might be preferable than other classical immunosuppressants (46). Another case report pointed out that repeated administration of infliximab for a certain period may be beneficial in the treatment of steroid-refractory CIP (47). However, after these treatments, there are still some cases that are reported to be deteriorating. Vickie et al. reported that a patient developed a diffuse alveolar hemorrhage and died of respiratory failure after high-dose corticosteroids, empiric antibacterial therapy, and infliximab (48). Recently, the success of triple combination therapy (high-dose corticosteroids, tacrolimus, and cyclophosphamide) for steroid-refractory CIP was reported (49). In addition to these traditional immunosuppressants, Filipe et al. proposed new perspectives to manage steroid-refractory CIP (50). They indicated that other anti-TNFα drugs (including etanercept, adalimumab, certolizumab, and golimumab) could be alternatives to infliximab and that anti-IL-1 therapy (anakinra or canakinumab) might be helpful for patients with severe anti-TNFα-refractory pneumonitis (50). Moreover, an anti-IL-6 (tocilizumab) strategy was also considered as an effective treatment option for steroid-refractory CIP (51). Nevertheless, further investigations are needed to seek a better management approach for steroid-refractory CIP.

For patients who suspend ICI treatment after CIP treatment, some of them can consider the rechallenge of ICIs. A pooled analysis collected 170 patients from 10 studies, 20 of whom developed CIP. Seven patients (35%) resumed treatment after suspending ICIs, and two patients developed CIP again and recovered after using steroids again (32). Patients receiving rechallenge should regularly evaluate the efficacy and closely monitor the adverse events, including CIP and other irAEs. If CIP relapses again, then no longer consider rechallenge after treatment.

In addition, empirical anti-infective treatment should be performed simultaneously if the cause of infection cannot be completely ruled out for G2∼G4 patients. For patients with more than 20 mg of prednisone (or equivalent doses) for >4 weeks, antibiotics should be considered for the prevention of pneumocystis pneumonia. When using glucocorticoids, clinicians are supposed to consider using proton pump inhibitors (PPIs) to prevent gastrointestinal reactions, and if using steroids for a long time, patients need to be supplemented with calcium and vitamin D.

## Mechanism of Checkpoint Inhibitor Pneumonitis and Translational Investigations

Currently, the mechanisms for CIP are poorly understood. 20 years ago, studies reported that PD-L1 or CTLA-4 gene-deficient mice developed multisystem autoimmune diseases including pneumonitis (52). Michael et al. pointed out several possible mechanisms of irAEs, including increased T cell activity, increased autoantibody levels, increased levels of inflammatory cytokines, and enhanced complement-mediated inflammation (3). The above expositions could explain the possible mechanisms of myocarditis, colitis, thyroiditis, and pituitary inflammation caused by immunotherapy with ICIs, but whether these mechanisms are responsible for CIP remains unknown.

### Which and How Do Immune Cells Play an Important Role in Checkpoint Inhibitor Pneumonitis?

Due to the lack of preclinical models, several studies focused on the patient’s BAL fluid (BALF) and lesion tissue to explore the underlying mechanisms of CIP.

Several studies have reported that an increased number of lymphocytes and a small number of eosinophils and neutrophils can be found in BALF of the patients with CIP (53–55). In an autopsy case, Koelzer et al. found that interstitial lymphocytic infiltration and fibrotic rings occurred between lung lobules, around the bronchioles and under the pleura, rich in CD8 + T cells, with high expression of PD-1 and cytotoxic granule-associated RNA binding protein (TIA-1) (56). Another research performed PD-L1 staining on lung biopsy tissue and found a large number of macrophages with high PD-L1 expression in the alveolar space (57). These findings indicate that T lymphocytes and macrophages may play a role in the occurrence and development of CIP.

A study analyzed the landscape of the immune cells in alveolar and found that proinflammatory subsets (central memory T cells, IL-1β^hi^ populations) increased and the anti-inflammatory process was inhibited (decreased expression of CTLA-4 and PD-1 in T regulatory cells and decreased expression of counter-regulatory interleukin-1 receptor antagonist) in both T cells and myeloid cells in BALF, providing the possible underlying mechanisms of immune dysregulation in patients with CIP (58). Another study compared the T cell clonality between the resected pneumonitis lesion and the primary tumor of a patient with CIP, finding that there is a clear overlap between them. Through the above research, the author suggests that one possible mechanism of CIP is that tumor-specific T cells via the blood circulation to the lung sharing antigens with the tumor result in the immune response in the patient (59). But whether these are tumor-infiltrating lymphocytes (TILs) requires further investigation.

### Is There a Key Cytokine, Chemokine, or Molecule?

Several animal studies indicated that PD-L2 played an essential role in the mechanisms of CIP. The expression of PD-L2 mainly concentrates on immune cells, such as dendritic cells (DCs), and Th2 cells, which belong to the subset of CD4 + T cells and can secrete Th2 cytokines (such as IL-4, IL-5, IL-10, and IL-13). As for non-immune cells, PD-L2 expresses in epithelial cells, especially lung epithelial cells. An animal experiment indicated that the PD-L2 could combine with the repulsive guidance molecule b (RGMb) secreted by lung interstitial macrophages and alveolar cells and could promote the increase of initial T cells that leads to respiratory immune tolerance (60). Anti-PD-1 agents could promote the combination of PD-L2 and RGMb by reducing the combination of PD-L2 and PD-1, thus leading to vigorous clonal expansion of lung resident T cells. At the same time, the PD-1 blockade would hinder the respiratory immune tolerance of this expanded clone and eventually led to immune-mediated toxicity in the lungs (61). In addition to RGMb, some scholars pointed out that the Th2 inflammation caused by the blockade of PD-1/PD-L2 interaction was also a possible mechanism of CIP (62). Moreover, IL-6 seems to play an important role in CIP, and it is considered to be a biomarker for irAEs, including indirect signs of high inflammation associated with IL-6, such as increased CRP (63). IL-6 was reported to function as a main cytokine in the generation of a cytokine release syndrome (CRS) and viral respiratory distress syndrome (ARDS) by stimulating T cell proliferation and affecting the ability of pulmonary DCs to prime naive T cells (64). And a study proved that anti-IL-6 could also be effective for CIP in addition to treating CRS (51). Nevertheless, it requires more basic researches to support and to explore the specific mechanism of IL-6 in the occurrence of CIP.

### Is There a Direct Result of Immune Checkpoint Inhibitor Drugs or a Combination of Factors?

Because the mechanisms of pneumonia are still poorly understood, it is unknown whether there are multiple factors involved in the occurrence of CIP. A previously combined underlying disease may be one of several factors, including asthma, COPD, and chronic/low-grade infection. Besides, other previous or ongoing cancer treatment may be another factor involved, including chemotherapies, targeted therapies, radiation therapy, or other immune therapies. Some other factors, such as active or passive exposure to cigarette smoke, different cancer types, and different ages, may also have an impact on CIP (6, 35, 65). In addition, in the checkmate078 study with the East Asian population as the main subject, the rate of overall lung toxicity induced by nivolumab was 7%, and in a phase II study in Japanese patients, the incidence was 8% (66, 67). In comparison, the rates in the checkmate017 and checkmate057 studies with the white population as the main subjects were 3% and 5%, respectively (67). The incidence of CIP in the eastern population seems to be a little bit higher than in the western population, but whether different races will affect the development of CIP still needs more data.

## Discussion

At present, the risk factors for CIP are not completely clear. Based on our current understanding of it, clinicians should focus on the patients who have a smoking history, previous radiotherapy, and baseline lung disease, prior TKI, etc. All patients treated with ICIs should be alert to the possibility of CIP when they have new respiratory symptoms or increased initial respiratory symptoms.

Due to the lack of specificity of the clinical manifestations and imaging features of CIP, the diagnosis of CIP is a diagnosis of exclusion, and there is no unified diagnostic standard. Clinicians need to make a comprehensive judgment based on the history of ICI medication, clinical manifestations, imaging features, and laboratory examinations. In patients with suspected CIP, the possibility of lung infection, tumor progression, interstitial pulmonary diseases caused by other causes, pulmonary vasculitis, and pulmonary edema, etc., needs to be ruled out.

Moreover, due to the lag and persistence of the immune response, CIP can occur at any time during the treatment process, even after the end of treatment. Therefore, the patient’s condition is supposed to be monitored and followed up throughout the survival time.

In addition, there exists a controversy about the relationship between the occurrence of CIP and the efficacy of immunotherapy. One possible reason is that these studies are retrospective, and the incidence of CIP is low, resulting in a small number of patients in the CIP group (the minimum is 3 and the maximum is 38). Besides, one of these studies pointed out that non-specific manifestations of lower grade CIP, such as fatigue, might lead to misclassification (5). In the future, prospective, multicenter, large-scale researches are still warranted to explore related issues. At the same time, it is necessary to strengthen the understanding of CIP and improve the accuracy of diagnosis.

For now, many confusing issues need to be clarified. At present, the pathogenesis of CIP remains at the stage of research on individual cases. To better understand the biological mechanism of CIP, the treatment of CIP patients and the results of various examinations (including chest CT, pulmonary function testing, blood routine, and liver and kidney function) should be accurately and completely recorded, and the preservation of specimens (including BAL, lung biopsy tissue, and blood) should be ensured. The above data and specimens can be used as the bases for translation studies. Besides, there is no CIP-related animal model, so the establishment and application of the experimental model are also one of the difficulties that need to be overcome. In the future, a large number of samples need to be systematically studied and summarized to provide a more comprehensive understanding of CIP. Additionally, more translational and basic research is urgently needed to understand the underlying mechanisms better.

## Author Contributions

SZ, YF, BoZ, BiZ, and JW analyzed the literatures and researches and drafted the manuscript. SZ and YF contributed equally to this work. All authors contributed to the article and approved the submitted version.

## Conflict of Interest

The authors declare that the research was conducted in the absence of any commercial or financial relationships that could be construed as a potential conflict of interest.
